# Arteriovenous fistulas at the craniocervical junction: a review

**DOI:** 10.3389/fneur.2026.1794484

**Published:** 2026-06-25

**Authors:** Xin Ding, Guanghao Zhang, Chenghao Shang, Yuhang Zhang, Xiangjun Xiao, Jingning Wang, Guoli Duan, Zhe Li, Qiang Li

**Affiliations:** 1 Neurovascular Center, Changhai Hospital, Naval Medical University, Shanghai, China; 2 Department of Neurology, Shanghai 411 Hospital, Shanghai, China

**Keywords:** angioarchitecture, arteriovenous fistula, craniocervical junction, endovascular treatment, microsurgery, subarachnoid hemorrhage, venous hypertensive myelopathy

## Abstract

Craniocervical junction (CCJ) arteriovenous fistulas (AVFs) are rare vascular malformations and are characterized by complex angioarchitecture and variable clinical presentation. The most common manifestations are subarachnoid hemorrhage and venous hypertensive myelopathy, with hemorrhagic presentation more often associated with ascending intradural or intracranial venous drainage, venous varices, aneurysmal structures, and spinal arterial feeders. Because the natural history remains incompletely defined, accurate angiographic characterization is essential for classification and treatment planning. Microsurgery remains the main treatment for most CCJ AVFs because of its high obliteration rate and durability, whereas endovascular treatment is useful in selected anatomically favorable lesions but may be limited by incomplete occlusion, recurrence, and ischemic risk in complex cases. Prognosis depends on presentation, lesion subtype, venous drainage pattern, age, baseline neurological status, timing of diagnosis, and treatment-related complications; hemorrhagic onset generally has a better outcome than venous hypertensive myelopathy. Structured angiographic follow-up remains important after treatment.

## 1 Introduction

The craniocervical junction (CCJ) is a critical and complex anatomical nexus (1). Arteriovenous fistulas (AVFs)—abnormal shunts between arteries and the venous system—occur throughout the neuraxis but are exceptionally rare at the CCJ, comprising only 1–2% of cranial or spinal AVFs (2–7). Their management is challenging because of unique angioarchitecture (8, 9). Although recent advances have improved diagnosis and treatment (7, 8, 10–16), the rarity and vascular complexity continue to complicate radiologic identification and neurosurgical planning (7, 10, 17, 18). This review summarizes CCJ anatomy and angioarchitecture, natural history, classification, treatment strategies, complications, and outcomes.

## 2 Anatomy and angioarchitecture

The CCJ lies between the skull base and upper cervical spine, formed by the occipital bone and the first two cervical vertebrae (C1, atlas; C2, axis), spanning the caudal brainstem and rostral cervical cord (5, 19). The transition between lower brainstem and upper cervical cord is at the level of the most rostral C1 rootlets (20, 21). Biomechanically, the CCJ comprises (1) a central pillar (central basiocciput, odontoid process, and C2 body) and (2) two osseous rings—the foramen magnum ring (lateral basiocciput, exocciput with occipital condyles, and opisthion) and the C1 ring (anterior/posterior arches with lateral masses) (22–24).

CCJ AVFs arise within this region (25). While some literature includes foramen magnum (FM) dural AVFs within “CCJ AVF” (5, 8, 10, 11, 13, 26–29), lesions at C1–C2 differ from FM dural AVFs despite shared venous pathways (29). The angioarchitecture of C1–C2 CCJ AVFs more closely resembles spinal dural AVFs with respect to fistulous configuration and arterial supply (8, 30). Accordingly, several authors restrict “CCJ AVF” to lesions at C1–C2 and exclude FM DAVFs and spinal AVFs at C3 or below (7, 14, 16, 17, 31, 32).

Principal arterial feeders arise from vertebral artery (VA) branches, including the anterior spinal artery (ASA), posterior spinal artery (PSA), radicular arteries, anterior/posterior meningeal arteries, and muscular branches (6, 7, 10, 33–35). In a large series, radiculomeningeal VA branches supplied 98% of lesions, and spinal arteries (ASA and/or PSA) contributed in 63% (7). Song et al. (16) reported the dural branch of the VA (DBVA) as the predominant feeder in 109 fistulas (66.1%). External carotid artery contributions—commonly via the ascending pharyngeal artery and occipital artery—also occur (31, 34, 36). Flow-related aneurysms may form on feeding arteries under hemodynamic stress (17, 37–39); Song et al. (16) found them in 57 fistulas (34.5%), with higher prevalence in perimedullary AVFs (PAVFs; *p* < 0.001).

Venous drainage is extensive and heterogeneous, encompassing extradural and intradural systems (20, 21, 40, 41). Extradural channels include interconnected paravertebral and epidural plexuses (33, 42); the paravertebral plexus communicates with the deep jugular vein, sigmoid sinus, and suboccipital plexus (20, 34, 43). Intradural drainage involves veins of the lower brainstem and upper cervical cord, including anterior/posterior median and lateral spinal veins with medullary anastomoses (17, 20, 21). Bridging veins convey flow from brainstem and upper cervical cord to adjacent sinuses or paravertebral/epidural plexus via radicular veins along nerve rootlets (17, 20). Most CCJ AVFs drain intradurally, typically ventral to the medulla or spinal cord (15). Physiologically, venous outflow from the upper cervical cord courses caudally via bridging and radicular veins to the lateral epidural plexus; with a CCJ AVF, elevated venous pressure can drive drainage far caudally (thoracic to sacral levels) and, via rich anastomoses, cranial reflux into the intracranial venous system. Overloaded outflow frequently recruits multiple venous pathways (6, 8, 10, 28, 32, 44–46).

In Ma et al. (47), all PAVFs drained into the paravertebral venous plexus, and drainage direction correlated with presentation (*p* < 0.001): ascending intradural and epidural drainage were more frequent in subarachnoid hemorrhage (SAH; *p* < 0.001 and *p* = 0.003), whereas descending intradural drainage predominated in venous hypertensive myelopathy (VHM; *p* < 0.001). Aneurysmal structures occurred in 60 patients (30.3%) and were more common in SAH (p < 0.001). Venous varices were present in 135 patients (68.2%) (47). Similarly, Song et al. (16) found varices in 108 fistulas (65.5%), with lower incidence in epidural AVFs.

## 3 Natural history

The natural history of CCJ AVFs is incompletely characterized. Available series show a predominance in middle-aged men (male:female ≈3:1) (10, 11). The two principal phenotypes are SAH and VHM (6–8). Compared with aneurysmal SAH, hemorrhage from CCJ AVFs is often less severe (11, 47). Other presentations include intramedullary hemorrhage, brainstem dysfunction, occipital neuralgia, and neck pain (2, 10, 13, 48, 49).

Clinical presentation correlates with venous drainage: descending intradural drainage is associated with VHM, whereas ascending intradural or intracranial drainage is linked to hemorrhage (8, 11, 32, 47, 50). In Hiramatsu et al. (7), hemorrhagic onset was most common (SAH 63%; intramedullary hemorrhage 10%). Other cohorts similarly report SAH as a frequent initial symptom (10, 11, 15, 47). Risk factors for hemorrhage include intracranial venous drainage, venous varix, feeder aneurysm, and ASA supply (7, 11, 12, 15, 47, 51). Varices of draining veins are common, approaching 80% in some series (32, 37). Epidural drainage is less often hemorrhagic.

In VHM, early venous hypertension–mediated cord dysfunction may be reversible; persistent congestion can culminate in venous infarction and permanent deficits (11). Anterior or posterior spinal/perimedullary venous drainage may increase VHM risk. Patients with VHM tend to be older, likely reflecting insidious onset, longer duration, and diagnostic delay (47, 52).

Corticosteroids often exacerbate spinal dural AVFs, including CCJ lesions, possibly via acute increases in venous hypertension from fluid retention, venous engorgement, impaired outflow, and thrombosis (2, 52–58). Thus, DAVF should be excluded before starting corticosteroids in patients with chronic progressive myelopathy (2, 55).

## 4 Imaging features

For screening, contrast-enhanced CTA and MRA are useful (32, 59–62). CTA may show dilated draining veins (5), while MRA (reported sensitivity ≈90%) aids localization by depicting early-filling veins and can guide subsequent DSA (60–63). DSA remains the diagnostic gold standard, best demonstrating spinal arteries and fistula feeders (5, 64–70). Imaging advances have improved anatomic understanding and may refine risk assessment (7, 71–74). Three-dimensional rotational angiography (3D-RA) with high-resolution MIP/MPR delineates microvasculature and its bony relationships (7, 73, 75, 76), and 3D-RA/3D-MR fusion enhances shunt-site localization and overall diagnostic accuracy (77).

## 5 Classification

Hiramatsu et al. (7) proposed a five-type CCJ classification based on feeders, presumed shunt location, and venous egress: (1) dural AVF (DAVF)—meningeal feeders, dural shunt, intradural venous drainage; (2) radicular AVF (RAVF)—radicular/meningeal feeders, shunt at spinal nerve roots, radicular venous drainage; (3) epidural AVF (EDAVF)—radicular/meningeal feeders with pial contributors, shunt outside the dura, epidural venous drainage; (4) EDAVF without pial feeders; (5) perimedullary AVF (PAVF)—spinal pial-artery feeders, shunt on the cord surface, intradural venous drainage. Conventional angiography often cannot pinpoint the shunt, leading to inter-observer disagreement (5).

**Figure 1:**
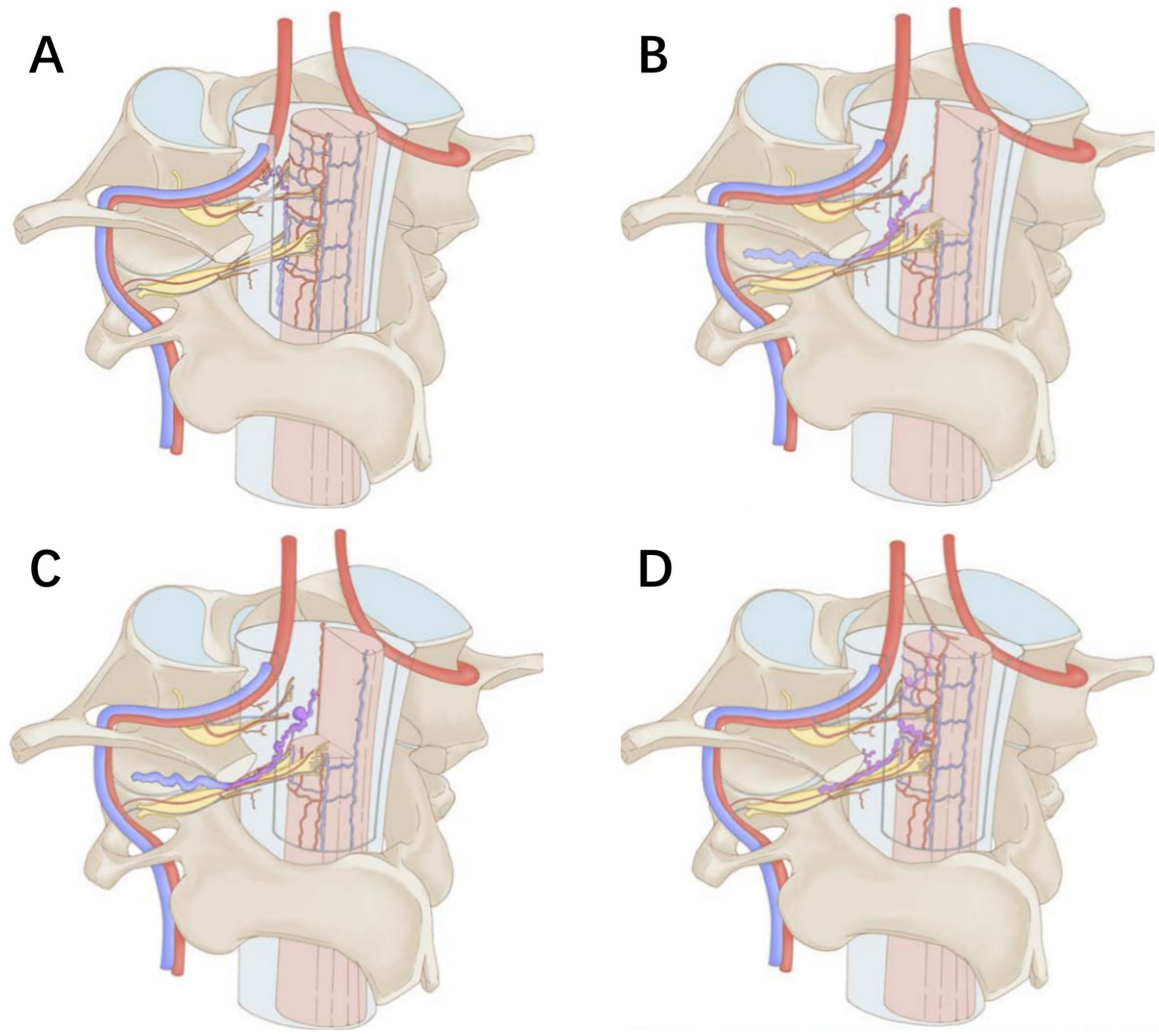
Schematic illustration of each type CCJ AVFs: **(A)** Dural AVF, **(B)** radicular AVF, **(C)** epidural AVF, **(D)** perimedullary AVF [collected from Song et al. (16)].

Song et al. (16) integrated digital subtraction angiography and intraoperative findings to categorize CCJ lesions into EDAVF (shunt external to dura), DAVF (dural shunt), RAVF (root shunt), and PAVF (cord-surface shunt). Across cohorts, DAVF is most frequent, followed by RAVF, EDAVF, and PAVF (7, 16, 47). CCJ DAVFs are typically dural-artery fed, dural based, and may show broad, irregular fistulous connections with upward or downward intradural venous outflow or lateral epidural drainage (5, 7, 31). RAVFs can resemble DAVFs and are frequently misclassified (7) but more often have dual dural-and-pial supply, are situated between dura and cord, and are usually supplied by spinal pial arteries—feeders uncommon in DAVFs (29). Clinically, DAVFs tend to cause venous congestive myelopathy, whereas RAVFs more often harbor fistulous-site aneurysms and draining-vein varices and may present with hemorrhage. In a Japanese multicenter series (54 patients, 59 lesions), RAVFs were more prevalent than expected, and hemorrhagic risk with RAVFs was 1.73-fold that of DAVFs (7). In Ma et al. (47), SAH was associated with higher proportions of EDAVF, PAVF, and RAVF (*p* = 0.048, 0.012, and 0.009), whereas VHM was more frequent in DAVF (*p* < 0.001). Song et al. (16) showed that RAVFs and PAVFs more commonly exhibited ascending intradural drainage (*p* < 0.001), DAVFs descending intradural drainage (*p* < 0.001), and EDAVFs predominantly epidural drainage (*p* < 0.001). EDAVFs can be subclassified by the presence of spinal pial feeders (7, 9). Lesions without pial supply or intradural drainage are generally benign (25), whereas EDAVFs with intradural reflux, marked venous congestion, or a pial-feeder aneurysm warrant aggressive treatment (29). Compared with other CCJ AVFs, EDAVFs may drain more widely (e.g., into the suboccipital plexus or posterior cervical veins) (29, 78). Most PAVFs are ventral or ventrolateral to the cervical cord (79), often receive multiple radiculomedullary and/or spinal pial feeders, drain directly to perimedullary veins (6, 7, 17, 31), and are typically high-flow with varices at the fistulous site or along the draining vein (25) (see Figure 1).

## 6 Treatment and complications

CCJ AVFs may present with acute hemorrhage, VHM, brainstem dysfunction, or radiculopathy; symptomatic or ruptured lesions warrant urgent intervention (37). Choice of therapy is driven primarily by detailed angioarchitecture (47, 80). Two principal modalities are used: microsurgical disconnection and endovascular treatment (EVT) (8, 10, 11, 15, 17, 32, 81–83). Historically—mirroring spinal AVFs—microsurgery has often been favored and is associated with higher cure rates (37, 81, 84, 85). In a systematic review of CCJ dural AVFs, complete obliteration was achieved in 98% after microsurgery versus 56% after EVT (11). Complications can occur after either approach, including ischemic and hemorrhagic events, hydrocephalus, and cerebrospinal fluid (CSF) leakage (8, 14, 17, 80). Given nontrivial procedural risks, Inoue et al. (86) advised caution with prophylactic treatment of asymptomatic CCJ AVFs; none of their asymptomatic cases became symptomatic during follow-up.

### 6.1 Microsurgery

#### 6.1.1 Microsurgical strategies

Microsurgery yields superior complete obliteration rates and remains first-line for most CCJ AVFs. The surgical corridor is determined by lesion location: posterior median for dorsal fistulas and far-lateral for lateral lesions (47, 80). For CCJ DAVFs and RAVFs with an intradural feeder, the primary goal is interruption of this feeder and its associated draining vein, typically via suboccipital craniotomy with C1 laminectomy. If no intradural feeder exists, the goal is disconnection of the intradural draining vein (6, 78, 87). For PAVFs, the aim is coagulation of the entire shunt complex, including all relevant feeders and drainers (31). Precise intraoperative identification of the fistula, feeding arteries, and draining vein—while preserving normal spinal arteries and veins—is essential (31, 78, 79).

Because the AVF site may not be obvious in the operative field, procedures should ideally be performed in a hybrid operating room to allow intraoperative DSA. Techniques such as occlusion testing and indocyanine green (ICG) videoangiography aid shunt localization and vessel confirmation (8, 31, 84). After disconnection, ICG angiography or intraoperative DSA is necessary to confirm complete obliteration (80). ICG videoangiography is a minimum requirement; where intraoperative angiography is unavailable, timely postoperative DSA is mandatory (16, 80). For ventrolateral lesions, exposure can be enhanced by mobilizing the cord via the dentate ligament or by table rotation. Fistulas within the ventral one-third of the cord are particularly challenging and are often accompanied by spinal pial-artery–fed aneurysms, making safe microsurgical clipping or ventral drainage disconnection nearly impossible (47).

#### 6.1.2 Microsurgical innovation

Endoscopic assistance has emerged as a useful adjunct for ventral CCJ AVFs and may improve access and visualization (9, 18, 79). Fistulas with spinal arterial feeders or predominantly ventral location (e.g., selected PAVFs and RAVFs) frequently exhibit complex architecture with aneurysmal structures (9, 47, 80). Whether such aneurysms/varices require direct occlusion is debated. These structures are likely flow-related (88). Takai et al. (15) showed that after treating the AVF alone, associated aneurysms/varices can regress spontaneously, suggesting a paradigm of treating the shunt first.

#### 6.1.3 Microsurgical efficacy and safety

Most series report favorable outcomes after microsurgery (7, 8, 11, 15, 80, 83). In Song et al. (80), microsurgery alone cured 98 of 122 fistulas (80.4%) in 113 patients. Takai et al. (15) reported that only 2 of 78 surgically treated patients (2.6%) required retreatment.

Complications include ischemic and hemorrhagic events, hydrocephalus, and CSF leakage (8, 14, 80). Reported rates range from 14 to 26%, higher with major hemorrhage at presentation or highly complex fistulas (8, 14, 47, 52, 80). Takai et al. (15) documented complications in 17 patients (22%). Ischemic events may result from inadvertent posterior spinal artery coagulation (14).

### 6.2 Endovascular treatment (EVT)

Despite its minimally invasive nature, EVT is not optimal for most CCJ AVFs (10, 11, 15, 89). Transarterial access is often limited by small-caliber, tortuous feeders arising at sharp angles from the VA (8), creating risk of iatrogenic perforation during microcatheter navigation (5, 90). EVT is therefore reserved for selected cases with simple angioarchitecture, such as a solitary feeder without pial contribution (6). For example, Alshekhlee et al. (91) obliterated a CCJ PAVF via a thick, straight ASA using coils. Many CCJ EDAVFs with intradural venous drainage can be effectively treated by transarterial liquid embolization; for EDAVFs with favorable venous access, transvenous embolization is a potential—though technically demanding—option (92).

Historically, EVT has had higher complication and recurrence rates than microsurgery. In Takai et al. (15), overall EVT complications were 42%; ischemic complications (spinal/brainstem infarctions) were 26%, more than triple the microsurgery rate—likely reflecting complex angioarchitecture (14, 15). Risk analysis identified interventional embolization (OR 4.3, 95% CI 1.1–16, *p* = 0.030) and spinal arterial feeders (OR 3.8, 95% CI 1.03–14, *p* = 0.045) as predictors of ischemic complications (14). Hazardous anastomoses between radiculomeningeal and radiculopial systems can permit unintended embolic penetration and compromise cord or brainstem perfusion (14, 15).

Advances in embolic materials and technique continue to improve EVT safety, enabling catheterization of smaller vessels and better depiction of angioarchitecture with superselective angiography (88). EVT thus retains a role in anatomically favorable CCJ AVFs and as an adjunct before definitive microsurgery (16).

## 7 Prognosis

Outcomes depend on presentation and lesion subtype. Hemorrhagic onset generally portends better recovery than VHM. At the CCJ, DAVFs more often manifest with venous congestion, whereas intradural RAVFs and PAVFs more frequently present with hemorrhage; accordingly, intradural/extradural AVFs often achieve better outcomes than DAVFs. In an untreated cohort, Li et al. (93) estimated a 15–20% risk of neurological deterioration, and VHM at presentation was associated with a 30% rate of neurological disability. Microsurgical series report favorable results in >90% after disconnection (6, 8, 12, 31, 32, 37). Younger age predicts better functional outcome (44, 94), and timely diagnosis facilitates immediate fistula obliteration with improved prognosis (11). Ma et al. (47) identified age ≥56 years, VHM at presentation, and pre-treatment mRS ≥ 3 as independent predictors of poor outcome. In Takai et al. (14), procedure-related complications were strongly associated with unfavorable outcome (OR 5.8; 95% CI 1.3–26; *p* = 0.020). These data underscore the need for early, precise treatment planning and meticulous delineation of feeders and venous drainage—particularly when spinal pial supply is present—to minimize complications.

## 8 Conclusion

Craniocervical junction arteriovenous fistulas are rare vascular shunts with complex angioarchitecture and heterogeneous clinical manifestations. Their presentation and prognosis are strongly influenced by lesion subtype and venous drainage pattern, particularly with respect to hemorrhage and venous hypertensive myelopathy. Accurate diagnosis requires detailed angiographic assessment, and treatment should be individualized according to angioarchitecture and clinical status. Microsurgery remains the primary treatment for most lesions, while endovascular therapy is appropriate in selected anatomically favorable cases. Overall prognosis is generally favorable after appropriate intervention, although delayed diagnosis and treatment-related complications remain important determinants of outcome.
